# Medical College of Ohio

**Published:** 1888-08

**Authors:** 


MEDICAL COLLEGE OF OHIO —See the advertisement of this excellent school in to-days RECORD.

